# Case Report: A rare case of long-term survival in primary pulmonary adenoid cystic carcinoma with bilateral renal and chest wall metastases

**DOI:** 10.3389/fonc.2026.1732593

**Published:** 2026-03-04

**Authors:** Chunlin Huang, Zhihua Ye, Xiaoyun Zhou, Junkai Zhang

**Affiliations:** 1 Guangdong Medical University, Zhanjiang, China; 2 Department of Pulmonary Oncology, Zhongshan City People’s Hospital, Zhongshan, China

**Keywords:** bilateral renal metastasis, case report, chest wall metastasis, lung cancer, primary pulmonary adenoid cystic carcinoma

## Abstract

Primary pulmonary adenoid cystic carcinoma (PACC) is an exceedingly rare malignant lung tumor. We report an extremely rare case of a 38-year-old female. In 2013, a computed tomography (CT) scan suggested lung cancer in the left upper lobe, and a percutaneous biopsy confirmed PACC pathologically. She underwent surgical resection followed by postoperative adjuvant chemotherapy in the same year, with no recurrence during the 5-year post-operative follow-up. In December 2019, follow-up CT revealed bilateral renal metastases. Subsequently, in 2021, chest wall metastases developed. After sequential radiotherapy, chemotherapy, and combined immunotherapy, the chest wall mass was significantly reduced. During this period, multiple immune-related adverse events (irAEs) occurred. Upon progression of the renal metastases in 2024, ultrasound-guided ablation was performed. Subsequent re-evaluations showed essentially no viability in the bilateral renal tumors, and the chest wall mass remained stable. This patient was diagnosed with PACC in 2013. As of September 2025, her overall survival (OS) has exceeded 11 years. The successful management of this case is attributed to multimodal therapy. To date, no cases of PACC with concurrent bilateral renal and chest wall metastases have been reported, thus providing a valuable reference for the diagnosis and treatment of PACC.

## 1 Introduction

Adenoid cystic carcinoma (ACC) is a malignant tumor that predominantly arises in the salivary glands; it is exceptionally rare as a primary lung malignancy, accounting for less than 0.2% of all primary lung cancers. PACC primarily originates from the peribronchial glands, and its morphology is similar to that of salivary gland ACC. It is characterized by slow progression and late distant metastasis, thus classified as a low-grade malignancy (1).

PACC predominantly affects younger patients. Factors including clinical stage at diagnosis, surgical margins, patient age, and growth pattern significantly influence prognosis. Patients diagnosed with early-stage PACC have a favorable prognosis. Tumor size and patient age are independent prognostic factors (2, 3). However, due to the rarity of PACC, there is a lack of large-sample clinical studies. Its biological characteristics are not fully understood, and its clinical behavior and optimal treatment remain unclear. Therefore, this article presents a case of PACC in which the patient’s disease course has exceeded 11 years from diagnosis. This case provides rare long-term follow-up data and successful experience with multimodal treatment, offering new insights for clinical practice. Written informed consent was obtained from the patient for this case report.

## 2 Case

### 2.1 Initial diagnosis and surgical treatment (2013)

**Figure 1:**
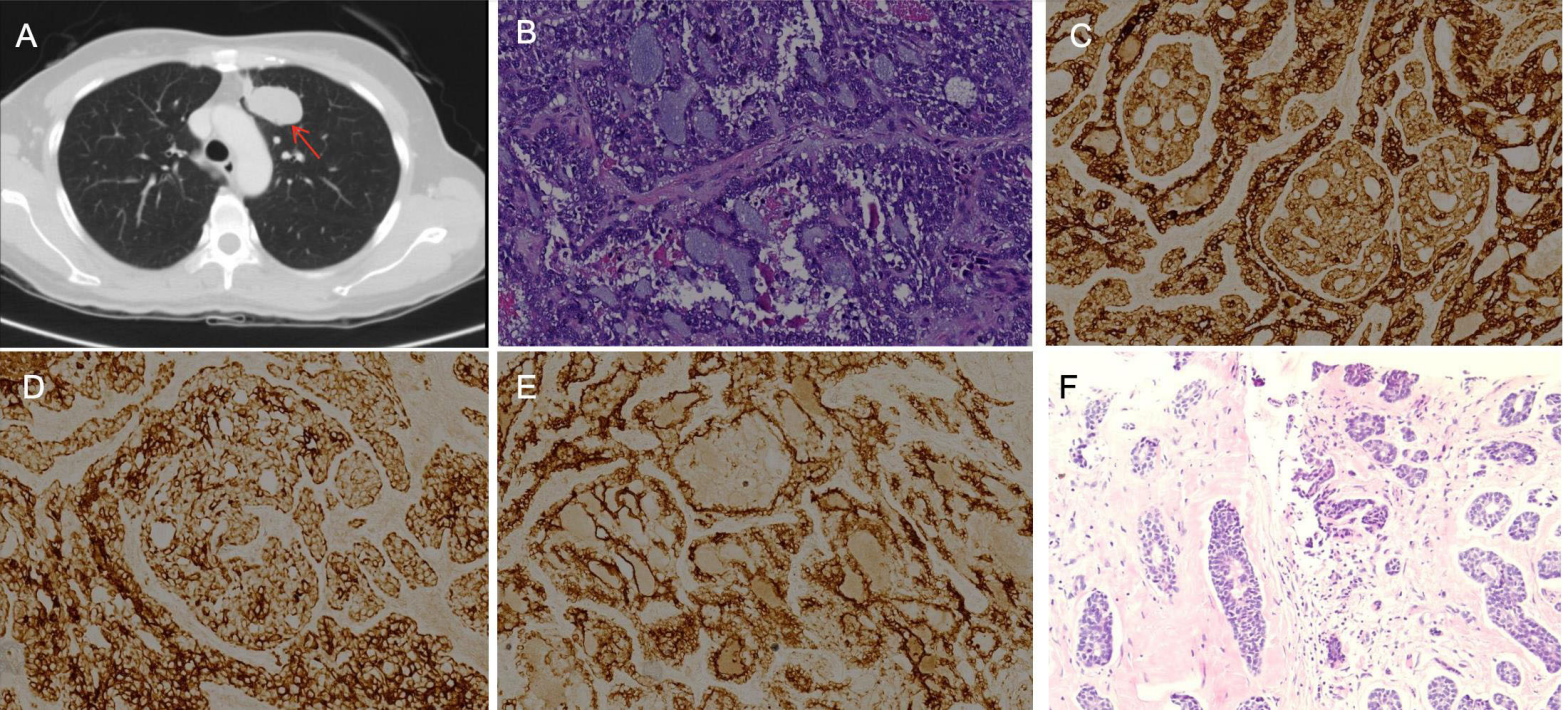
Imaging and pathological images: **(A)** The lung CT scan shows a peripheral lung cancer in the upper left lung. **(B)** The postoperative lung tissue pathological image (HE×200) is PACC. IHC×400 shows **(C)** CK (+), **(D)** CK7 (+), **(E)** EMA (+). **(F)** The puncture of the chest wall mass confirmed metastatic ACC (HE×200).

A 38-year-old female patient presented in August 2013 with an unprovoked cough, without obvious clinical symptoms such as dyspnea or chest pain. Her past medical history was unremarkable. She denied a smoking history or family history of lung cancer. The patient’s Eastern Cooperative Oncology Group Performance Status (ECOG PS) was 1. CT revealed a peripheral lung carcinoma in the upper lobe of the left lung, measuring approximately 35×22 mm (**Figure 1A**). In September 2013, a percutaneous lung biopsy confirmed the diagnosis of pulmonary adenoid cystic carcinoma pathologically. In October of the same year, she underwent video-assisted thoracoscopic left upper lobectomy. Postoperative pathology was consistent with adenoid cystic carcinoma: the maximum tumor diameter was approximately 3 cm, with no evident lymphovascular invasion, negative surgical margins, and no lymph node metastasis. Immunohistochemistry (IHC): CK7+, CK+, Vimentin+, TTF-1+, CK5/6 partially+, Syn-, chromogranin A focally+, P63+, calponin+, SM-actin+/-, CEA+, S-100+, EMA+ (**Figures 1B–E**). Polymerase chain reaction (PCR) testing for EGFR mutations was negative. She was staged as pT2aN0M0 (IB) per the 7th edition of the AJCC Cancer Staging Manual. She received 2 cycles of adjuvant chemotherapy with paclitaxel liposome (210 mg, day 1) and nedaplatin (40mg, days 1-3). No recurrence or metastasis was observed during the 5-year postoperative follow-up.

### 2.2 Discovery of bilateral renal metastases (2019)

**Figure 2:**
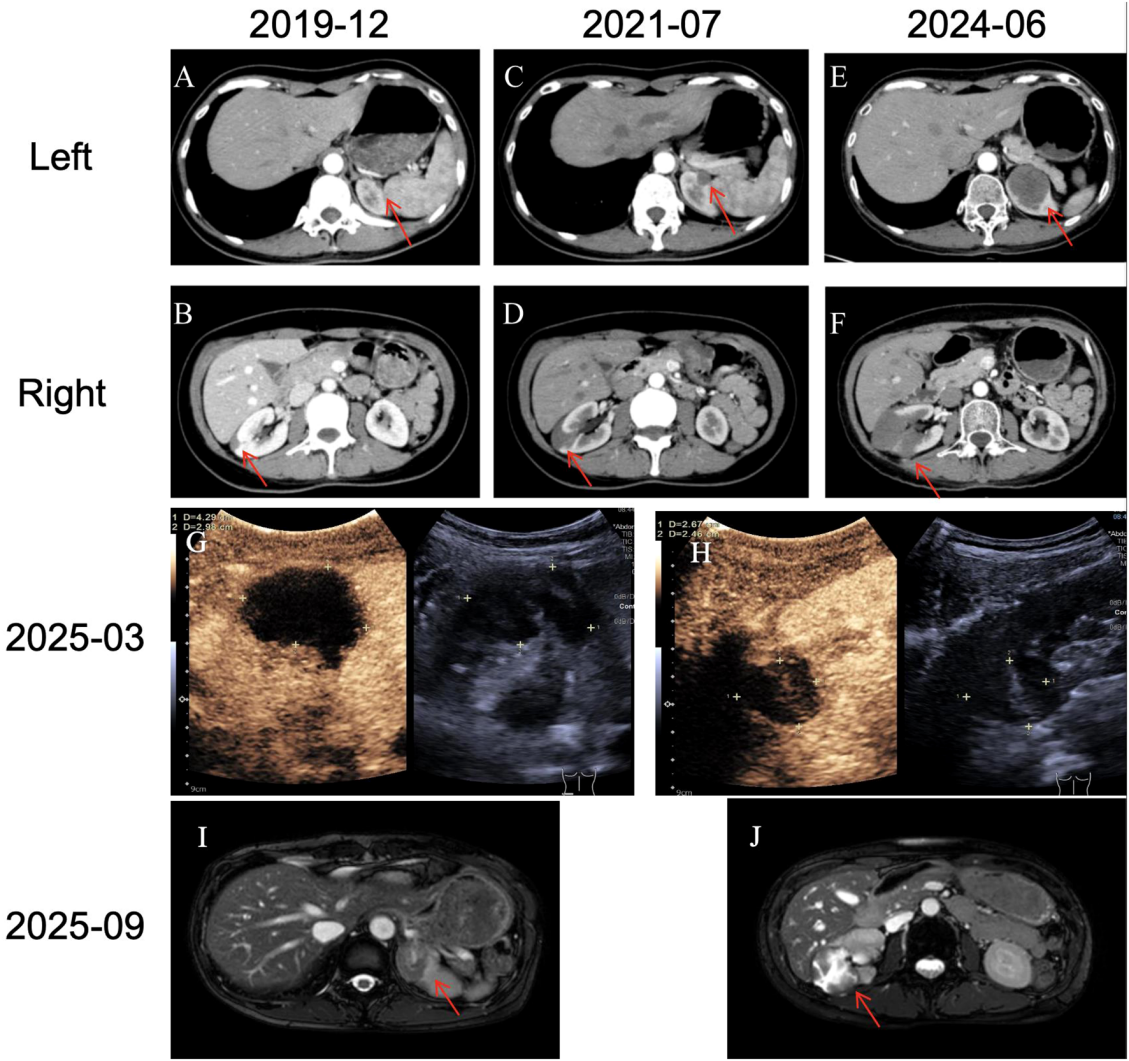
Follow-up images of renal metastases. **(A-F)** The changes in the location and size of bilateral renal masses on imaging. Contrast-enhanced ultrasound of both kidneys: **(G)** 27×25mm on the left side; **(H)** Two masses on the right side, the larger one being 27×25mm. No enhancement was observed on both sides. **(I, J)** MR suggested changes in bilateral renal masses after treatment, with no obvious activity.

In December 2019, during a routine follow-up, the patient’s repeat chest CT scan first identified bilateral renal metastases: the largest lesion was located subcapsular in the right kidney, measuring 14×8 mm (**Figure 2B**), and the left renal metastasis measured 7×5 mm (**Figure 2A**). Before chemotherapy, an echocardiogram was performed and no abnormalities in the heart structure were found. In December 2019, chemotherapy with liposomal paclitaxel (220 mg, day 1) in combination with lobaplatin (40 mg, day 1) was initiated. Due to severe adverse effects like vomiting, the regimen was changed to pemetrexed monotherapy (0.7g, day 1) for 1 cycle in January 2020. Chemotherapy was discontinued due to poor tolerance. From January 2020 to June 2021, the patient discontinued anti-tumor therapy due to personal reasons. However, during this period, two CT re-examinations indicated that the size of the bilateral kidney metastases remained stable.

### 2.3 Comprehensive treatment following discovery of chest wall metastasis (2021-2023)

**Figure 3:**
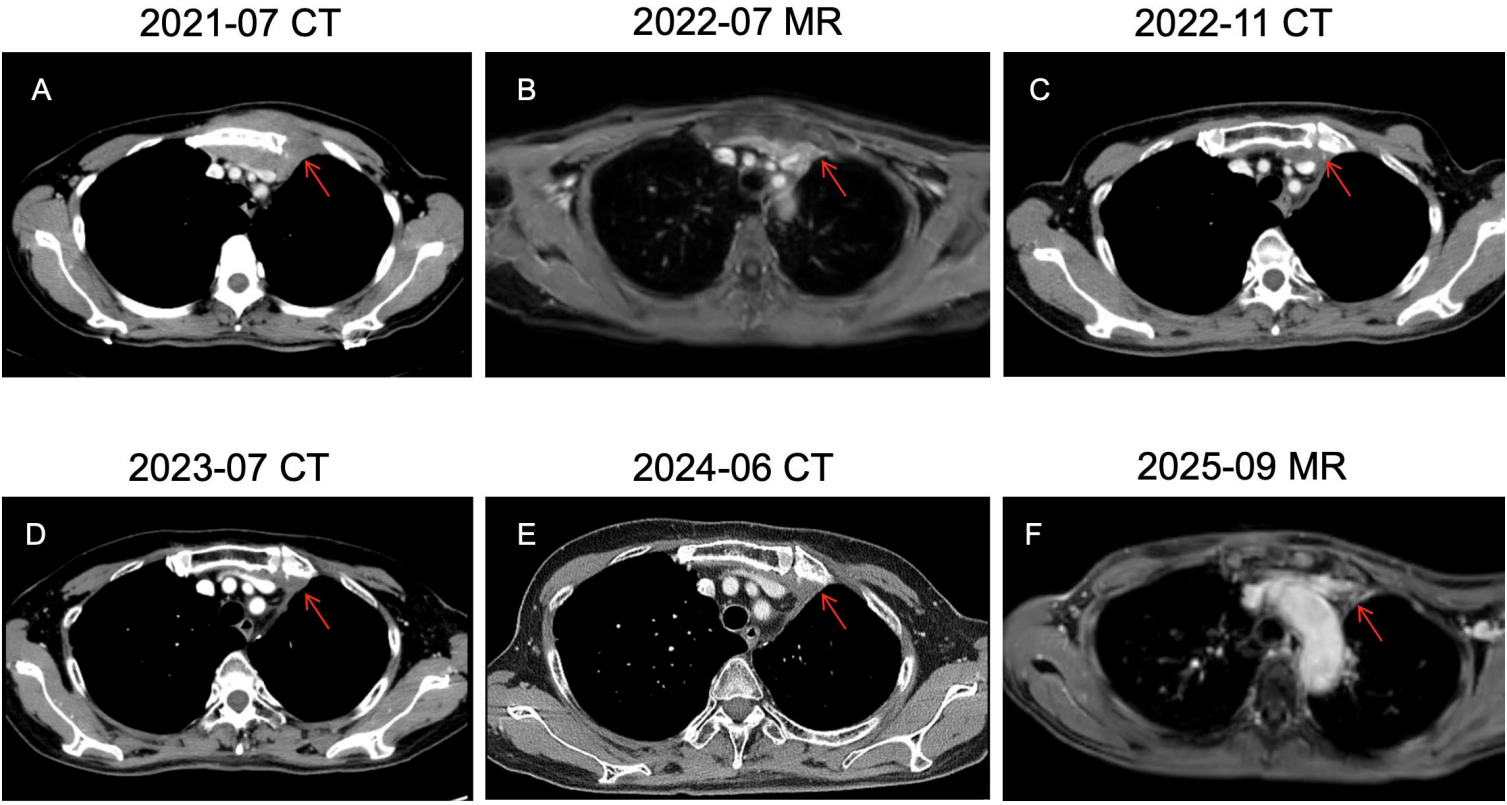
**(A-F)** Follow-up images of chest wall metastases, showing the dynamic changes of the metastatic masses in the chest wall.

In July 2021, a painless mass appeared on the chest wall and enlarged rapidly. Contrast-enhanced chest CT revealed a parasternal soft tissue mass with a maximum cross-sectional dimension of approximately 81×40 mm (**Figure 3A**). Biopsy pathology confirmed metastatic ACC (**Figure 1F**). Bilateral renal metastases had increased in size compared to before, with the right one measuring 20×10mm and the left one 11×10mm (**Figures 2C, D**). Per the Response Evaluation Criteria in Solid Tumors (RECIST) version 1.1, the treatment response was assessed as progressive disease (PD). The chest wall tissue specimens were sent to Shanghai DaAn Laboratory for high-throughput gene sequencing. The results showed that no clinically significant gene mutations related to the indications for tumor-targeted therapy or tumor specificity were detected. EGFR and MYB-NFIB gene mutations were also negative. The tumor mutation burden (TMB) was 1.4 mutations per megabase, and the microsatellite status was stable (MSS). From July to September 2021, she received 4 cycles of docetaxel (90 mg, day 1) and nedaplatin (40mg, days 1-3) combined with bevacizumab (600mg, day 1), with efficacy evaluated as stable disease (SD). From October 2021, treatment with the programmed cell death 1 (PD-1) inhibitor sintilimab (200 mg, day 1) combined with capecitabine (1 g, days 1-14) was administered until February 2022. Subsequent magnetic resonance imaging (MR) showed slow enlargement of the chest wall tumor, with the largest cross-section approximately 90×34 mm (**Figure 3B**). From March 2022, the regimen was changed to sintilimab (200 mg, day 1) combined with vinorelbine (80 mg, once weekly) and anlotinib (10 mg, days 1-14). Re-evaluation indicated SD. In May 2022, radiotherapy (RT) was delivered to the chest wall metastasis (45 Gy/15 fractions). Immunotherapy was paused during RT, while vinorelbine and anlotinib were maintained concurrently. A CT scan performed in November 2022 demonstrated a significant reduction in the size of the chest wall metastatic tumor compared to prior imaging, with the maximum thickness measuring 15 mm. The therapeutic response was assessed as partial response (PR) (**Figure 3C**). This patient received long-term immunotherapy. During the treatment period, no cardiac-related symptoms such as chest pain or palpitations occurred. The results of regular serum troponin tests were all within the normal range, and no structural abnormalities were found in the echocardiogram.

### 2.4 Occurrence and management of irAEs (2023-2024)

In May 2023, the patient presented with oral blisters accompanied by blisters on the waist and other areas. Physical examination revealed scattered blisters all over the body, with clear boundaries and a suspected positive Nikolsky’s sign. Laboratory tests showed C4 0.46g/L, C-reactive protein (CRP) 5.8 mg/L, and C3 1.57g/L. Pathological biopsy suggested the possibility of pemphigus (**Supplementary Figure 1**). IHC indicated IgG (+), while IgA, IgM, C3, and Fib were all (-). The dermatology department considered it drug-induced pemphigus related to immune checkpoint inhibitors (ICI). According to the NCI Common Terminology Criteria for Adverse Events (NCI-CTCAE) version 5.0, it was graded as grade 2. Methylprednisolone (32 mg, once daily) improved significantly after two weeks of glucocorticoid therapy. Since we could not rule out anlotinib as the cause of the rash. We considered the increased risk of combination therapy and the potential benefit of immunotherapy for the patient. Anlotinib was permanently discontinued, while sintilimab was continued. In January 2024, laboratory tests indicated a cortisol level of 19.3 ng/ml, leading to a diagnosis of immune checkpoint inhibitor-related adrenal insufficiency, for which steroid replacement therapy was initiated. In February 2024, the patient’s examination showed ALT 227 U/L, AST 485 U/L. The diagnosis was ICI-related hepatitis grade 3. The initial treatment was prednisone (30 mg, once daily). The dosage was then gradually reduced to 10 mg once daily based on the patient’s condition. Follow-up reexamination showed that ALT dropped to 27 U/L and AST dropped to 30 U/L. However, after discontinuing prednisone, the liver enzyme levels rebounded. Therefore, long-term maintenance treatment with oral prednisone 10 mg once daily was required, and the immunotherapy was permanently discontinued thereafter. The levels of liver enzymes and cortisol, along with their longitudinal follow-up trends, are illustrated in **Supplementary Figure 2**.

### 2.5 Local treatment for renal metastases and current status (2024-2025)​

A CT scan in June 2024 showed local progression of the bilateral renal metastases, evaluated as progressive disease (PD). The left renal mass measured 37 × 36 mm (**Figure 2E**), and the right renal mass measured 23 × 21 mm (**Figure 2F**). The patient had a long-standing history of antineoplastic drug therapy and had developed severe treatment-related adverse reactions. Given the patient’s poor tolerance, the regimen was switched to a weekly schedule. In July 2024, weekly paclitaxel liposome 60 mg and cisplatin 30mg were administered, but the patient developed Grade 3 hepatotoxicity, leading to chemotherapy discontinuation. In October and November 2024, ultrasound-guided microwave ablation (MWA) was sequentially performed for the patient’s bilateral renal metastases. For both ablation sessions, the parameters were set as follows: power of 55 W, duration of 5 minutes, and a monopolar electrode was employed without water isolation. No post-procedural complications were observed. A follow-up contrast-enhanced ultrasound of the kidneys in March 2025 indicated no activity in the bilateral solid renal nodules (**Figures 2G, H**). In September 2025, MR showed that the metastatic tumors in both kidneys had no obvious enhancement (**Figures 2I, J**). The chest wall mass had significantly shrunk (**Figure 3F**). The treatments received by the patient at each stage and related events are shown in **Table 1**. As of September 2025, the patient’s OS exceeds 11 years. The patient continues to be under follow-up.

**Table 1:** Treatment schedule encompasses defined time intervals, therapeutic regimen, efficacy assessment, and treatment-emergent adverse events.

Date	Treatment/Clinical interventions	Response evaluation	Major events/Treatment-related adverse events
2013.10 - 2014.02	Thoracoscopic left upper lobectomy. Two cycles of adjuvant platinum-based chemotherapy were performed.	R0 resection	Remained disease-free for 5 years
2019.12 - 2020.01	Liposomal Paclitaxel 220 mg D1 + Lobaplatin 40mg D1. →Pemetrexed monotherapy 0.7 g D1.	SD	Bilateral renal metastases with severe nausea and vomiting occurred after chemotherapy.
2020.01 – 2021.06	Temporary suspension of anti-tumor therapy	SD	Treatment was held due to personal reasons.
2021.07 – 2021.10	Docetaxel 90 mg D1 + Nedaplatin 40 mg D1 + Bevacizumab 600 mg D1 for 4 cycles.	PD→SD	Chest wall metastasis was identified, showed progressive enlargement on imaging.
2021.10 – 2022.04	Sintilimab 200 mg D1 + Capecitabine 1 g Bid→Sintilimab 200 mg D1 + Vinorelbine 80mg Qw + Anlotinib 10 mg D1-14	SD	The chest wall metastasis remained stable during the combined treatment.
2022.05	Radiotherapy for chest wall metastases(45Gy/15 fractions)+ Vinorelbine 80mg Qw + Anlotinib 10 mg D1-14	Not evaluated	During radiotherapy, immunotherapy was suspended to avoid the superimposition of toxicities.
2022.07 – 2023.04	Vinorelbine 80mg Qw + Anlotinib 10 mg D1-14	PR	CT showed over 50% reduction in the chest wall metastasis and stable renal metastasis.
2023.05 – 2024.02	Long-term glucocorticoid therapy was administered, and the use of anlotinib and sintilimab was discontinued.	Not applicable	irAEs: Drug-related pemphigus(G2) Adrenal insufficiency(G3) Hepatitis(G3)
2024.06 – 2024.07	Dose-modified chemotherapy:Paclitaxel liposome 60 mg Qw + Cisplatin 30 mg Qw	PD	Hepatic insufficiency (G3) after chemotherapy
2024.10 – 2024.11	Bilateral renal metastases, MWA	PR	Imaging findings indicated the disappearance of activity in bilateral renal metastases.
2024.12 – now	Regular follow-up	SD	The condition remains stable.

## 3 Discussion

ACC is a biphasic tumor composed of ductal and myoepithelial cells, exhibiting three histological patterns: cribriform, tubular, and solid. The cribriform pattern is the most common, while the solid pattern is the most aggressive and prone to metastasis (4, 5). The cribriform pattern features characteristic sieve-like spaces containing hyaline and mucoid material. Molecularly, ACC pathogenesis has been strongly linked to the MYB-NFIB fusion and/or MYB overexpression (6). Notably, high-throughput sequencing of this patient failed to detect MYB gene mutations, which may reflect the biological heterogeneity of the tumor. In this case, HE staining confirmed the histological morphology as the cribriform pattern (**Figure 1B**).

The organs most commonly involved in metastatic ACC are the lungs, bones, brain, and liver (7). The prognosis for PACC patients with distant metastasis varies, and the distant metastases often share similar growth characteristics with the primary tumor. Renal metastasis is considered a rare phenomenon. Most patients are asymptomatic at the time of renal metastasis diagnosis. Based on limited data, the median time to renal metastasis appearance is approximately 13 years, and it is usually unilateral (8). Currently, studies on renal metastasis of PACC are still limited to a few retrospective case analyses. A retrospective study of adrenocortical carcinoma with renal metastasis (n = 10) reported a median time to metastasis of 13 years. Among the 8 patients available for follow-up, survival outcomes showed marked heterogeneity: 6 patients maintained long-term disease control and stable survival, while the other 2 patients died 3 and 4 years after detection of renal metastasis, respectively (9). A similar case reported bilateral renal metastases that developed 14 years after the initial diagnosis of PACC (10). Following surgical resection of these metastases, the patient remained free of recurrence or distant metastasis 18 months after surgery (11). The occurrence of PACC with simultaneous bilateral renal and chest wall metastases, as in this case, has not been previously reported.

Surgery remains the primary treatment modality for PACC. However, the tendency for perineural invasion and infiltrative growth in ACC often limits the feasibility of complete resection. Therefore, for unresectable or incompletely resected tumors, postoperative adjuvant radiotherapy and/or chemotherapy are optimal choices (12). Regarding systemic therapy for PACC, there are no specific guideline recommendations. Current treatment approaches generally refer to guidelines for non-small cell lung cancer (NSCLC). Following the detection of metastasis, the patient was administered chemotherapy regimens including paclitaxel, platinum-based agents, and pemetrexed sequentially. However, treatment was discontinued due to chemotherapy intolerance, and the therapeutic response during chemotherapy was not significant. Reports indicate that specific chemotherapy regimens have not shown significant efficacy in clinical trials, possibly due to the indolent biology of ACC (13). Objective response rates (ORR) vary widely across studies investigating chemotherapy for ACC, but platinum-based chemotherapy remains the first-line choice. Common regimens include cisplatin + vinorelbine (PV), carboplatin + paclitaxel, and cisplatin + doxorubicin + cyclophosphamide (CAP) (14). The PV regimen has an ORR of approximately 44.4%, while the CAP regimen has an ORR ranging from 25% to 33% (15).

The efficacy and safety of ICI monotherapy and ICI-combined chemotherapy for NSCLC have been well-established in numerous clinical trials. However, the data regarding its application in PACC is extremely limited. In this case, PD-1 inhibitor sintilimab, in combination with other therapies, resulted in a progression-free survival (PFS) of 28 months. During the phases combined with chemotherapy and targeted therapy, the best response was SD. However, after the addition of radiotherapy, the best response improved to PR. Notably, the chest wall tumor continued to shrink over the two years following radiotherapy completion, suggesting a potential synergistic effect between immunotherapy, targeted therapy, and radiotherapy. In ACC, PD-L1 expression is often negative or low, and TMB levels are also low (16). A study involving 27 cases of PACC, only 4 cases showed positive expression of PD-L1 on immune cells, which also confirms the above statement (17). The overall ORR for immune checkpoint inhibitors in ACC ranges between 0% and 8.7%, with median PFS between 4.4 and 8.3 months (14, 18). Therefore, immunotherapy is generally not recommended as a first-line treatment. While combination immunotherapy is a hotspot in oncology, safety concerns are significant. A phase II clinical study showed that when using nivolumab-ipilimumab for metastatic/recurrent ACC from any anatomical origin, 100% of patients experienced any-grade irAEs, and 45.8% experienced Grade 3–4 treatment-related adverse events (TRAEs) (19). In NSCLC, treatment with Nivolumab-Ipilimumab combined with two cycles of chemotherapy yields a superior OS compared to the pembrolizumab plus chemotherapy cohort. However, the incidence of TRAEs reaches 60%, indicating a need for further evaluation of the safety profile of dual immunotherapy (20). Most current studies on PD-1 inhibitors focus on a 2-year treatment cycle, with limited reports on longer durations. Research by Lova Sun indicated that among patients with advanced NSCLC receiving immune checkpoint inhibitors for over 2 years, 50% experienced immune-related adverse events (21). In this case, the patient developed multiple irAEs, including Grade III events requiring long-term oral glucocorticoids, after over 2 years of immunotherapy. This suggests that the onset of irAEs in such patients might be more delayed compared to other lung cancer types, highlighting the need for extended monitoring in PACC patients on prolonged immunotherapy and providing an important reference.

The patient received the multi-target tyrosine kinase inhibitor anlotinib for 14 months, with the best response evaluated as SD. This aligns with the results of a study on anlotinib in metastatic ACC, which reported a disease control rate (DCR) of 63.2% and a median PFS of 10 months, similar to the outcomes observed here (22). In the present case, anlotinib was administered in combination with sintilimab. A small-sample (n=22) phase IB study demonstrated that the sintilimab-anlotinib combination regimen yielded an objective response rate (ORR) of 72.7%, DCR of 100%, and PFS of 15 months in patients with NSCLC (23). Patients with chemotherapy intolerance may derive benefit from this combination regimen. Current targeted therapy for ACC primarily involves vascular endothelial growth factor receptor (VEGFR) inhibitors, but overall efficacy is limited. A retrospective analysis encompassing 17 studies showed that VEGFR inhibitors for recurrent or metastatic ACC yielded a 6-month DCR of 54%, an ORR of only 6%, and SD in 82% of patients (24), confirming their limited effectiveness.

Following local RT for the chest wall metastasis and MWA for renal metastases, the patient achieved a PR. RT is widely used in tumor therapy and is the most effective cytotoxic therapy for solid tumor patients (25). Although there are no unified guidelines for radiotherapy in PACC, data from studies on ACC in other sites suggest that radiotherapy, particularly stereotactic body radiotherapy, provides significant PFS and OS benefits for patients with oligometastatic lung lesions from ACC (26), offering a reference for local treatment in PACC. Additionally, MWA has proven efficacy and safety for small renal tumors (27), consistent with the results achieved in this case. Other studies have also demonstrated good local control of ACC metastases in the lungs and liver using local ablation techniques, providing a basis for local treatment of oligometastatic sites in PACC (28–30).

The unique biology of PACC necessitates individualized and multidisciplinary treatment plans. The multimodal synergistic therapy in this case resulted in a significant long-term survival benefit. Since the diagnosis of PACC in 2013, the patient has achieved an OS of 11 years, which substantially exceeds the levels reported in existing literature, providing an important paradigm for the long-term management of PACC. Chest wall metastasis and bilateral renal metastases are extremely rare sites for PACC spread, with no similar cases reported to date, making this a valuable dataset for future research. Future directions for PACC treatment should focus on further exploring its molecular biology and optimizing multimodal strategies to reduce toxicity and enhance efficacy.

## 4 Conclusion

This case details a rare instance of PACC with metastases to both kidneys and the chest wall. It highlights the importance of multidisciplinary management and the complexity of managing treatment-related toxicities, particularly given the rare metastatic sites and extended survival period. It provides significant reference for clinical decision-making. Although this study provides several insights into the treatment of PACC, its conclusions are mainly based on case reports and still need to be verified through larger sample surveys and independent cohort studies.

## Data Availability

The original contributions presented in the study are included in the article/**Supplementary Material**. Further inquiries can be directed to the corresponding author.
