# Supplementary figures and images for: Case Report: A rare case of long-term survival in primary pulmonary adenoid cystic carcinoma with bilateral renal and chest wall metastases
Source: Front Oncol. 2026 Mar 4;16:1732593. doi: 10.3389/fonc.2026.1732593 (PMC12995776; doi:10.3389/fonc.2026.1732593)

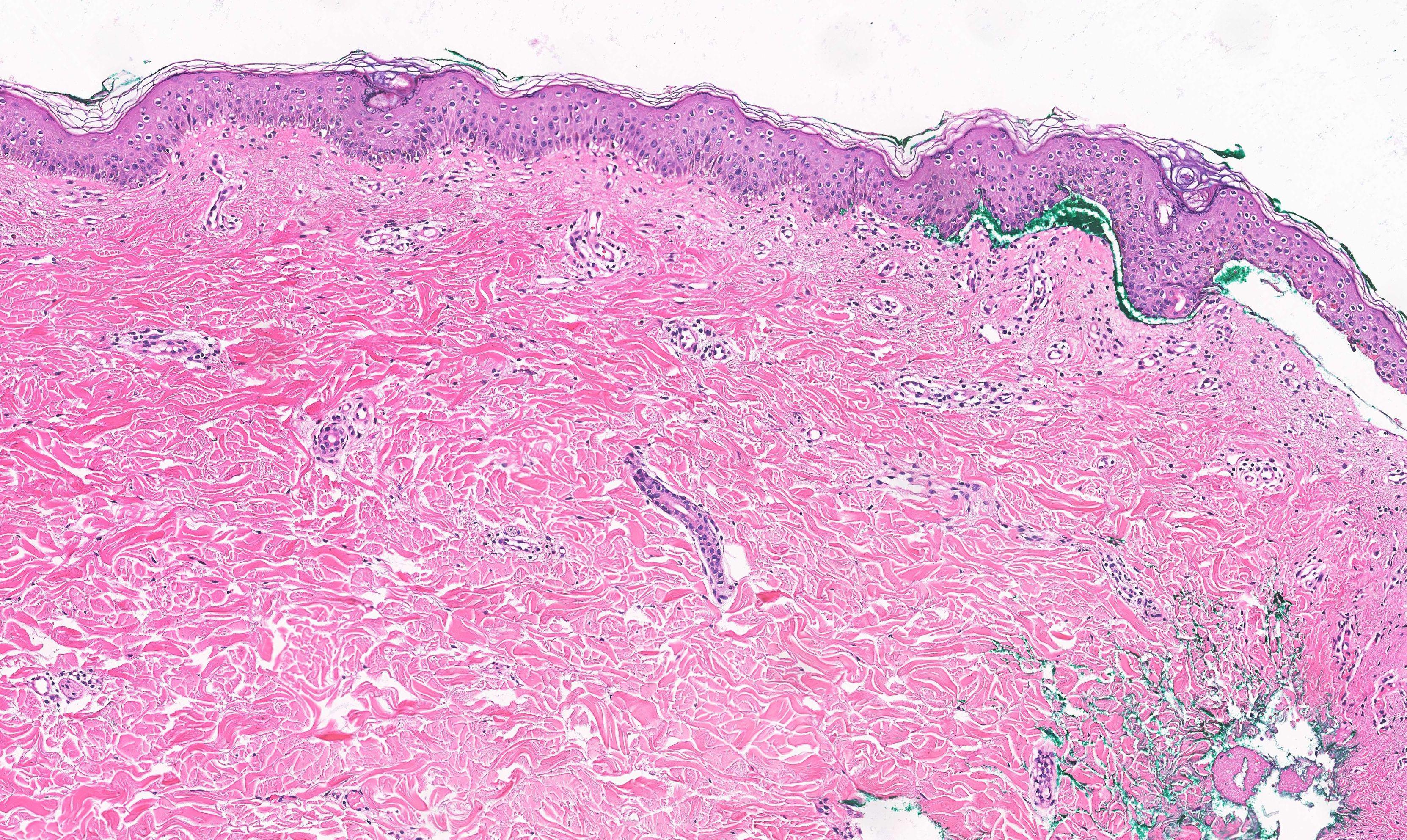

Supplement: Supplementary file 1 [file Image1.jpeg]
